# Possible Ambiguity in Interpretation Between Immunoglobulin G4-Related Disease (IgG4-RD) and Sarcoidosis in a Post-COVID-19 Pandemic

**DOI:** 10.7759/cureus.38124

**Published:** 2023-04-25

**Authors:** Rajesh Gurunathan, Pavithra Dhanasekaran, Dedeepiya Devaprasad, Sheba SK Jacob, Babu K Abraham

**Affiliations:** 1 Department of Acute Internal Medicine, Royal Stoke University Hospital, University Hospitals of North Midlands NHS Trust, Stoke-on-Trent, GBR; 2 Department of Critical Care Medicine, Apollo Hospitals, Chennai, IND; 3 Department of Geriatric Medicine, Royal Stoke University Hospital, University Hospitals of North Midlands NHS Trust, Stoke-on-Trent, GBR; 4 Department of Pathology, Apollo Hospitals, Chennai, IND

**Keywords:** igg4 and covid 19 infection, steroid responsive, elevated serum igg4, sarcoidosis “like, igg4- related disorders

## Abstract

A 36-year-old lady presented with fever, cough, maculopapular rash, painless sialadenitis, episcleritis, and arthralgia of more than 10 months, occurring in episodes since she tested positive for COVID-19 in 2020. Her symptoms were well controlled with corticosteroid and immunosuppressant therapy. Her clinical presentation and findings on bronchoscopy resembled that of sarcoidosis. However, the bronchial biopsy histopathology ruled out sarcoidosis. An increased serum immunoglobulin G4 level and its possible association with COVID-19 raises the question of whether the possibility of immunoglobulin G4-related disease (IgG4-RD) could be entertained.

## Introduction

Immunoglobulin G4-related disease (IgG4-RD) is a rare disorder affecting multiple systems that can mimic malignancy, infections, and inflammatory conditions [1]. Diagnosis mainly relies on clinical and histopathological features, including raised serum immunoglobulin G4 (IgG4) level [1,2]. The hallmark histopathological findings include a dense lymphoplasmacytic infiltrate, a storiform pattern of fibrosis, and obliterative phlebitis [1-3]. IgG4-RD typically responds well to glucocorticoids and immunosuppressant therapy [1]. Recent studies also demonstrated that people with IgG4-RD are more susceptible to COVID-19 infection, and COVID-19 vaccines increase the risk of relapse in people with IgG4-RD. This report describes the spectrum of symptoms a young lady with no known co-morbidities had experienced after getting infected with the coronavirus. The investigations and her presentation raised a clinical suspicion of whether she could have an atypical presentation of IgG4-RD.

## Case presentation

**Figure 1:**
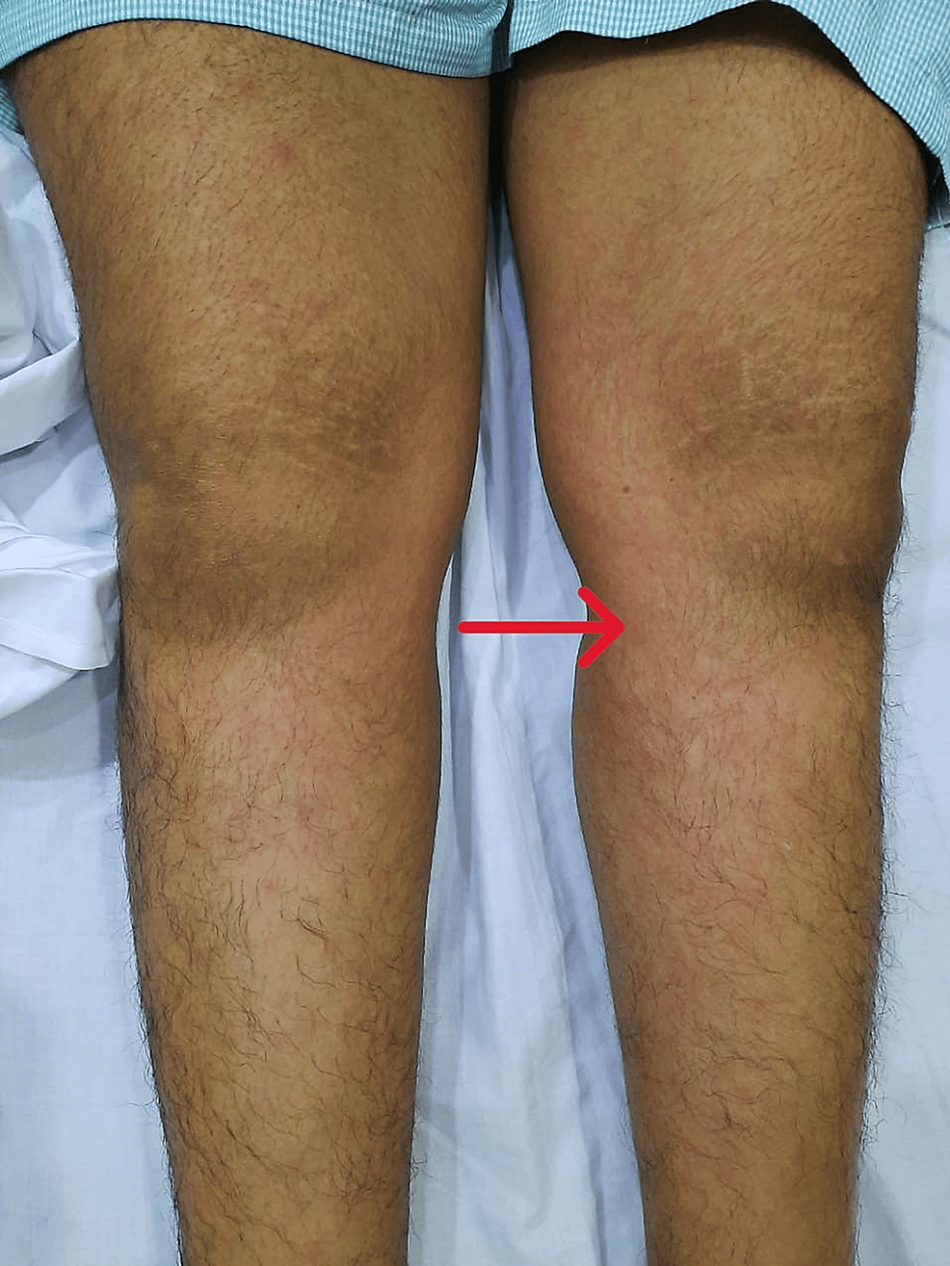
Maculopapular rash involving both lower limbs.

**Figure 2:**
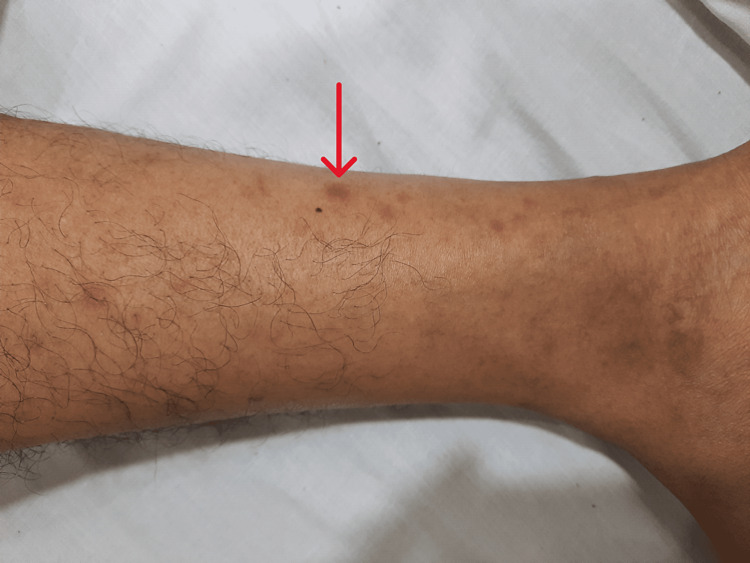
Maculopapular rash involving left leg.

A 36-year-old lady with no known co-morbidities tested positive for COVID-19 for the first time on August 14, 2020. She had flu-like symptoms for a few days and got better. She had no family history of any autoimmune conditions or malignancies. In mid-November 2020, she started having signs of upper respiratory tract infection (URTI), including a runny nose, sneezing, and low-grade fever. She consulted an otolaryngologist and was diagnosed with sinusitis with turbinate hypertrophy. Despite taking an oral antibiotic (cefpodoxime proxetil) and antihistamines for three weeks, her symptoms did not settle. She was advised to have a CT scan of her paranasal sinuses, and a diagnosis of pansinusitis was made. She was prescribed steroids, with which her symptoms got well controlled. However, her symptoms recurred on tapering the dose of steroids. From the first week of December 2020, she started having intermittent episodic high-grade fever and maculopapular rash (Figures 1-2) associated with chills and profound myalgia. She experienced 4-5 episodes in a month, lasting 2-3 days. By January 2021, she developed difficulty chewing, facial swelling, and periorbital puffiness associated with preauricular, postauricular, and submandibular lymphadenopathy. Initially, her symptoms were mild, and a high-resolution CT scan of the neck performed was normal. She received her first dose of the AstraZeneca Covishield vaccine on January 22, 2021. Her symptoms worsened soon after the COVID-19 vaccination. A neck ultrasound scan showed bilateral parotid enlargement with altered echotexture and cervical lymphadenopathy. She was prescribed antibiotics again (co-amoxiclav and amikacin) for five days, along with analgesics, which controlled her symptoms. From the last week of February 2021, her fever episodes were associated with arthralgia, for which she took symptomatic treatment. Her symptoms resolved gradually over the next 2-3 months.

**Figure 3:**
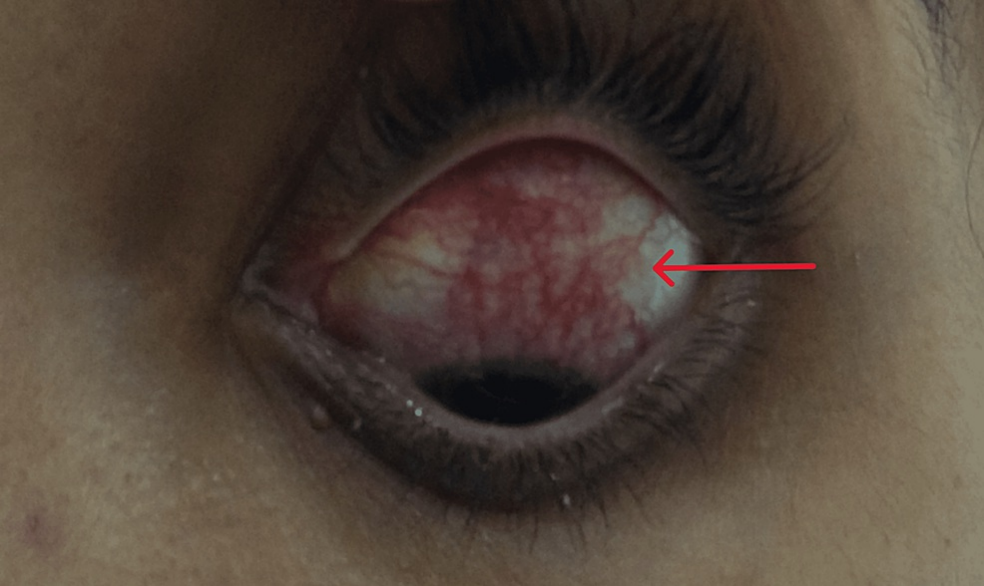
Episcleritis involving the left eye.

In mid-March 2021, she developed purulent nasal discharge and nose block, for which she was prescribed cefpodoxime for 10 days, along with a nasal decongestant spray and antihistamine. By the end of March 2021, she started to experience nasal crusting and a non-productive cough. A nasal swab grew Staphylococcus aureus, for which she received co-amoxiclav again for five days. Despite her nasal symptoms subsiding, her cough worsened, and she started having high-grade fever and rash episodes. During this period, she had multiple episodes of high-grade fever, severe irritant dry cough, arthralgia, and non-blanching rash (Figure 1), especially over the extremities, abdomen, and lateral aspect of the thigh. On examination, she was found to have a widespread maculopapular rash (Figures 1-2), especially on her trunk and extremities, which was pruritic, episcleritis in her left eye (Figure 3), few scattered crackles bilaterally on auscultating the lung fields. All other systemic examinations were unremarkable. 

Laboratory investigations are presented in Table 1. As summarized, serum IgG4 was markedly elevated. C-reactive protein, erythrocyte sedimentation rate, plasma fibrinogen, fibrin degradation product, and D-dimer were also elevated. Epstein-Barr nuclear antigen (EBNA) IgG and EBV VAC/EA IgG were found to be positive, serum calcium was just below the normal reference range, serum angiotensin-converting enzyme was normal, and absolute eosinophil count was elevated. Peripheral blood smear showed normocytic normochromic cells with neutrophilia, and bone marrow biopsy showed normocellular with trilineage hematopoiesis. 

**Table 1:** Relevant biochemical, hematological, and immunological investigations. EBNA IgG: Epstein-Barr nuclear antigen immunoglobulin G antibody; EBV VCA IgM: Epstein-Barr virus viral capsid antigen immunoglobulin M antibody; EBV VCA/EA IgG: Epstein-Barr virus - Viral capsid antigen/Early antigen immunoglobulin G.

Name and type of the investigation	Patient’s result	Reference range
Biochemistry		
C-reactive protein	165 mg/L	< 5.0 mg/L
Lactate dehydrogenase (LDH)	219 U/L	125-220 U/L
Serum angiotensin-converting enzyme (ACE)	31 U/L	8-52 U/L
Serum calcium	8.4 mg/dL	8.6-10.2 mg/dL
Absolute eosinophil count	Elevated	
Inorganic phosphate	3.9 mg/dL	2.5-4.5 mg/dL
Haematology		
Erythrocyte sedimentation rate (ESR)	70 mm/hour	0-20 mm/hour
Plasma fibrinogen	648 mg/dL	171-446 mg/dL
Fibrin degradation product	5 - 10 mcg/mL	<5 mcg/mL
D-dimer	2.65 mcg/mL	<0.5 mcg/mL
Activated partial thromboplastin time	31.7 seconds	27-34 seconds
Prothrombin time	14.4 seconds	12-16 seconds
Immunology		
Serum immunoglobulin G4 (IgG4)	386 mg/dL	2-121 mg/dL
Immunoglobulin profile		
Immunoglobulin A (IgA)	397 mg/dL	80-350 mg/dL
Immunoglobulin G (IgG)	1740 mg/dL	700-1600 mg/dL
Immunoglobulin M (IgM)	174 mg/dL	37-286 mg/dL
Epstein-Barr Virus (EBV) panel		
EBNA IgG	Positive	
EBV VCA IgM	Negative	
EBV VCA/EA IgG	6.46	0.10-0.21

Other investigations done included blood cultures, bone marrow culture, and aspiration cytology. Serum (1-3) beta-D-glucan, Aspergillus antigen- Galactomannan assay, anti-neutrophil cytoplasmic antibody (ANCA), anti myeloperoxidase (MPO) IgG antibody, anti proteinase 3 (PR3) IgG antibody, anti-glomerular basement membrane (anti-GBM) IgG antibody, anti-nuclear antibody (ANA), anti-double stranded DNA (ds/nDNA), extractable nuclear antigen IgG (Sm, Sm/RNP, SSA-Ro, Ro-52, SSB-La, Scl70, Jo-1), complement C3 and C4, anti streptolysin O titer, dengue serology (IgM and NS 1 AG), thyroid function tests (free T3, free T4, and thyroid-stimulating hormone) were performed, all of which were negative. Positron emission tomography-computed tomography (PET-CT) scan showed bronchial wall thickening involving the right and left bronchi and their proximal segmental branches with mild luminal narrowing. A fibro-optic bronchoscopy showed endobronchial mucosal thickening with diffuse pinpoint nodules scattered over bronchial segments involving all lobes. Bronchial wash cultures showed no acid-fast bacilli, and X-Pert Mycobacterium tuberculosis complex/resistance to rifampin (MTB/RIF) was undetected. Bronchial biopsy histopathology showed mucosa lined by respiratory epithelium and detached fragments of serofibrinous exudates with hemorrhage and few neutrophils and lymphocytes with no evidence of definite granulomas. A Mantoux test was negative. Histopathology of skin biopsy showed granulomatous vasculitis with associated fibrin thrombi in the dermal blood vessels. Immunofluorescence on skin biopsy was negative for IgG, IgM, IgA, C3c, C1q, and fibrinogen. Based on the above clinical and investigatory findings, differential diagnoses considered were (1) Sarcoidosis: endobronchial mucosal wall thickening and diffuse nodules on bronchoscopy and bronchial wall thickening on PET-CT; (2) IgG4-RD: strong association with COVID-19 and elevated levels of IgG4.

During her hospital stay, she was treated with antipyretics and IV antibiotics. She was also commenced on oral corticosteroids, to which she showed a dramatic clinical response. She was discharged home on a tapering regimen of glucocorticoid, starting at prednisolone 40 mg for two weeks with a plan to taper the dose every two weeks. During her first follow-up in July 2021, she made a remarkable recovery and returned to her routine activities. Once the prednisolone was tapered to a maintenance dose of 20 mg, she was started on Mycophenolate mofetil. However, within a month of tapering the dose of prednisolone, her symptoms started to recur, especially her cough. Increasing the dose of prednisolone to 50 mg while maintaining Mycophenolate mofetil at 2 grams brought her symptoms back under control. In October 2021, she developed avascular necrosis of her knee joint, and the prednisolone dose had to be tapered quickly. In late December 2021, her symptoms recurred and continued to progress in intensity till late January 2022. During this time, she tested positive for COVID-19 for the second time. In March 2022, she was given two injections of Rituximab, 1 gram each, following which her prednisolone was tapered to 10 mg, and Mycophenolate mofetil continued at 2 grams. Since then, her symptoms have remained controlled, and she was followed up till November 2022.

## Discussion

IgG4-RD is an uncommon fibro-inflammatory disorder affecting any organ, including the eyes, salivary glands, lungs, skin, heart, pancreas, and bile ducts [1]. Previously, it was presumed that IgG4-RD is related to type 1 autoimmune pancreatitis; however, recent studies show that IgG4-RD could manifest a spectrum of symptoms by affecting extra-pancreatic foci as well [1]. It is diagnosed with characteristic histopathological features, including dense lymphoplasmacytic infiltrate, storiform fibrosis, obliterative phlebitis, and, in some cases, moderate eosinophilia along with increased IgG4 + plasma cells in the tissue specimen [1-3]. Patients with IgG4-RD show increased serum immunoglobulin G4 levels; however, its concentration remains normal in up to 40% of people with biopsy-evident IgG4-RD [2]. It is estimated that 27%-53% of people with IgG4-RD have salivary gland manifestations [3]. Submandibular glands are commonly affected, but rarely parotid, sublingual, and labial salivary glands can be involved [3]. The clinical presentation shows bilateral, painless, persistent sialadenitis for over three months [3,4]. The prevalence of lung manifestations in IgG4-RD has been reported [4]. In a cross-sectional study of 114 patients with IgG4-RD, 14% were found to have pulmonary involvement [5]. Different patterns of pulmonary and intrathoracic manifestations, including nodules and masses in the parenchyma, nodular lesions in the pleura, oedematous bronchial mucosa, bronchial wall thickening, mediastinal and hilar lymphadenopathy, all of which are similar to those seen in sarcoidosis, have been reported in IgG4-RD [5]. Skin lesions are multifarious and caused by direct infiltration of IgG4+ plasma cells and IgG4-mediated inflammatory reactions [6,7]. In a study of 52 people with cutaneous IgG4-RD with 23 control cases, skin lesions were mainly observed as nodules (40.4%), papules (36.5%), and plaques (32.7%) [6]. These lesions are commonly located in the head and neck region (73.1%) and are less evidenced on the trunk (38.5%) and extremities (28.9%), and over 60% of these cases reported pruritus [6]. Recent studies demonstrate that COVID-19 infection and COVID-19 vaccines increase the chances of relapse in people with IgG4-RD [8,9]. Furthermore, the resemblance of cutaneous IgG4-RD to cytokine-mediated vasculitis after COVID-19 infection and vaccination is to be considered [10-12].

It is reported that IgG4-RD and related manifestations when diagnosed early, respond well to high-dose corticosteroids. Treatment with low-maintenance dose corticosteroids may be continued for up to three years to prevent relapse [1]. Immunosuppressants, including methotrexate, azathioprine, and mycophenolate, are the other drugs used to control the symptoms. However, their efficacy is still questionable [1]. This patient presented with a widespread non-blanching rash, episcleritis, irritant non-productive cough, and arthralgia. Her symptoms responded well to steroids and immunosuppressant therapy. Her clinical presentation, raised immunoglobulin G4 level, and the association of IgG4-RD with COVID-19 infection and vaccination make diagnosing IgG4-RD highly possible.

## Conclusions

This is a case of a steroid-responsive disease that has been a diagnostic challenge. Increased IgG4, eosinophilia, and IgG4-RD with COVID-19 raise the possibility of this being IgG4-RD. However, the lack of classical histopathological findings prevents confirmation of the diagnosis. Similarly, clinical signs and symptoms are akin to sarcoidosis, but the lack of histopathological confirmation practically ruled out the condition. In all likelihood, this could be an atypical presentation of IgG4-RD.
